# The Antibacterial Efficacy of Biopure MTAD in Root Canal Contaminated with *Enterococcus faecalis*

**DOI:** 10.5402/2012/390526

**Published:** 2012-08-01

**Authors:** Blerim Kamberi, Donika Bajrami, Miranda Stavileci, Shuhreta Omeragiq, Fatmir Dragidella, Ferit Koçani

**Affiliations:** ^1^Department of Dental Pathology and Endodontics, University Dentistry Clinical Center of Kosovo, 10000 Prishtina, Kosovo; ^2^Department of Microbiology, Directory of Water High Quality, Prishtina, Kosovo; ^3^Department of Periodontology and Oral Medicine, University Dentistry Clinical Center of Kosovo, 10000 Prishtina, Kosovo

## Abstract

*Aim*. The purpose of this in vitro study was to assess the antimicrobial efficacy of Biopure MTAD against *E. faecalis* in contaminated root canals. *Materials and Methods*. Forty-two single rooted extracted human teeth were inoculated with *E. faecalis* and incubated for four weeks. The samples were divided in two control and five experimental groups irrigated with 1.5% sodium hypochlorite solution (NaOCl); 3% NaOCl; BioPure MTAD; 1.5% NaOCl/17% EDTA; or 3% NaOCl/17% EDTA. After a one-week incubation, complete disinfection was confirmed by the absence of turbidity in the incubation media. Dentin shavings were taken from samples with no turbidity to verify whether *E. faecalis* was present in dentin tubules. Results were analyzed statistically using Fisher's exact test, with the level of significance set at *P* < 0.05. *Results*. Statistical analysis of the data obtained at Day 7 and after dentin shaving analysis showed that BioPure MTAD had significantly greater antibacterial activity than 1.5% NaOCl, 1.5% NaOCl/17% EDTA and 3% NaOCl/17% EDTA. No significant difference was detected between MTAD and 3% NaOCl. *Conclusions*. These findings suggest that BioPure MTAD possesses superior bactericidal activity compared with NaOCl and EDTA against *E. faecalis*.

## 1. Introduction

The major cause of endodontic failure is the survival of microorganisms in the root-filled tooth. Numerous authors have identified *E. faecalis *as the predominant microorganism found in root-treated canals displaying persistent periapical disease [1, 2]. The difficulty in eliminating *E. faecalis* from the root canal is due to its ability to adapt to environmental changes while retaining its pathogenicity [3]. Previous studies report a prevalence of *E. faecalis* ranging from 24–77% in teeth with failed endodontic treatment [4–8].

Endodontic infections are currently treated by mechanical debridement followed by chemical disinfection. Irrigants are used during the endodontic treatment to flush out loose debris, lubricate the dentinal walls, dissolve organic matter in the canal, and provide antimicrobial activity [9]. Sodium hypochlorite (NaOCl), at concentrations between 0.5–6%, is the most popular irrigating solution due to its antimicrobial activity and its ability to dissolve necrotic tissue [10]. However, sodium hypochlorite does not disinfect the entire root canal system, does not remove the smear layer from the dentinal walls, and is highly destructive when it comes into contact with the periapical tissues and gingiva [11]. In contrast, ethylenediaminetetraacetic acid (EDTA) has low or no antibacterial activity, but effectively removes the smear layer by affecting the inorganic component of the dentine. By facilitating the removal of infected tissue, EDTA contributes to the elimination of bacteria in the root canal [12]. Thus, to facilitate root canal disinfection, it is recommended that an irrigant containing both NaOCl and EDTA be used [11].

Torabinejad et al. [13] have reported the development of new irrigants for use in canal disinfection and smear layer removal, including BioPure MTAD (Dentsply, Tulsa, OK), a mixture of a tetracycline isomer [doxycycline], an acid [citric acid], and a detergent [Tween 80]. The doxycycline present in MTAD has high binding affinity for dentine, allowing for a prolonged antibacterial effect [14]. BioPure MTAD has been recommended as a final rinse irrigant because of its antimicrobial properties and its ability to remove the smear layer [11, 13, 15–17]. It is also less cytotoxic than most endodontic medicaments, including eugenol, hydrogen peroxide (3%), EDTA, and calcium hydroxide paste [15–18]. However, it has also been reported that BioPure MTAD (Dentsply) may not be effective against *E. faecalis* [19–21]. Therefore, the purpose of this study was to assess the antimicrobial efficacy of BioPure MTAD against *E. faecalis* compared to conventional endodontic irrigants. 

## 2. Materials and Methods

The methodology used in the present study was modified slightly from that described previously by Shabahang and Torabinejad [16]. Forty-two single-rooted extracted human teeth were used for this study. Samples were stored in water to avoid dehydration before use. After gaining access, the pulp was removed, irrigated with distilled water and all teeth were sterilized in an autoclave at 121°C (Sterilizatoren GmbH, Oiching, Germany).

Pure cultures of *E. faecalis* (ATCC 29212 OXOID, Hampshire, UK) were incubated overnight in blood heart infusion broth (BHI-Oxoid LTD., Wade Road, Basingstoke, Hampshire, UK) with a preparation of 1 × 10^8^ bacteria in 125 mL broth sufficient to prepare five experimental tubes. Teeth were immersed in inoculum and incubated at 37°C for four weeks in aerobic conditions in an incubator (INNOVENS 53, Jouan, France). This incubation period was sufficient for *E. faecalis* to invade the dentinal tubules. Culture media was refreshed every third day to maintain bacteria levels. Each tooth was sampled with paper points, from external and internal surface, inoculated on BHI plates to confirm the presence of infection.

Following four weeks of infection, samples were divided into seven groups: two control and five experimental groups. Working length was established by using #10 K-file to penetrate the apical foramen and then pulled back 1 mm. The teeth were manually instrumented with Flexo Files (Dentsply, Maillefeller Ballaigues, Switzerland) up to size #40 using a passive step-back technique. During cleaning and shaping, rigorous aseptic techniques were followed using sterile gloves and pliers. In the positive control group, irrigation was performed using distilled water. In the negative control group, the irrigant was also distilled water, but irrigation was followed by a period of autoclaving. Sterilization of these samples ensured that contamination of the working field did not occur. The remaining teeth were divided into five experimental groups, as detailed in Table 1.

Following preparation, each tooth was immersed in 2 mL of BHI and vortexed for 15 s. Teeth were then transferred into tubes containing fresh BHI and incubated for one week at 37°C under aerobic conditions. Complete disinfection was confirmed by the absence of media turbidity at a one-week period. Samples that showed turbidity were classified as infected, and the presence of bacteria was identified on BHI agar plates, and observed with microscope to identify Gram- positive cocci in chains. 

To determine the presence of bacteria in the dentinal tubule, dentin shavings were taken with sterile carbide bur no. 3. Shavings were taken from those samples that exhibited no turbidity to verify whether *E. faecalis* was present in the dentinal tubules. The teeth and dentin shavings were cultivated to determine the presence or absence of *E. faecalis*. Turbidity in cultivated solutions indicated the presence of *E. faecalis*. Shavings were collected on BHI agar plates and incubated for 48 h.

Results were analyzed by Fisher's exact test, with the level of significance set at *P* < 0.05.

## 3. Results

Turbidity was evident in all of the positive control samples, but in none of the negative controls. The presence of turbidity in test samples is summarized in Table 2. After one week of incubation, turbidity was evident to some degree in each of the groups treated with NaOCl or NaOCl + EDTA. In contrast, none of the samples treated with BioPure MTAD were visibly infected. When dentine shavings were incubated in BHI broth, a single sample in each of the five groups was infected with *E. faecalis*.

Statistical analysis of the total number of infected samples in each group using Fisher's exact test showed significant differences between BioPure MTAD and 1.5% NaOCl, 1.5% NaOCl/17% EDTA and 3% NaOCl/17% EDTA (*P* = 0.008 for each comparison), but no significant difference between BioPure MTAD and 3% NaOCl (*P* = 0.242). Because each group had a single sample (out of six) found to be infected in the dentinal tubules, this difference can be attributed entirely to differences in the rate of infection after the one-week incubation. Indeed, Fisher's exact test found near-identical results when analyzing these rates specifically (*P* = 0.008 for BioPure MTAD versus 1.5% NaOCl, 1.5% NaOCl/17% EDTA, 3% NaOCl/17% EDTA; *P* = 0.227 for BioPure MTAD versus 3% NaOCl.

## 4. Discussion

*Enterococcus faecalis* is commonly found in failed root-treated canals [22], due mainly to its resistance to chemo-mechanical procedures [6] and intracanal medication such as calcium hydroxide [23]. In our experiments, positive control samples showed that distilled water is totally ineffective at eliminating *E. faecalis*. Furthermore, negative control samples confirmed that the incubation environment used during the experiment was not contaminated. We chose several irrigants to evaluate their efficacy in eliminating *E. faecalis *from the root canal system. NaOCl, in addition to its excellent anti-bacterial properties, is known to be cytotoxic at higher concentrations, so we evaluated this irrigant at both low (1.5%) and high (3%) concentrations. Our results demonstrate that irrigation with NaOCl, even at the high concentration, eliminated *E. faecalis* in only half of the samples. This lack of efficacy of NaOCl in consistently disinfecting root canals is in agreement with results from previous investigations. A clinical study by Sjörgren et al. [24] concluded that 40% of root canal systems remain infected following irrigation with 0.5% NaOCl. In another study, SiqueiraJr et al. [10] investigated the ability of 4% NaOCl solution, used in various irrigation protocols, to eliminate *E. faecalis* from the root canal system and found that after irrigation with 4% NaOCl 30–40% of root canal systems remained infected with *E. faecalis*.

The smear layer is known to impede the penetration of antimicrobial agents into dentinal tubules [25]. We therefore included Groups D and E that were irrigated with 1.5% and 3% NaOCl, but were additionally treated with 17% EDTA prior to the final incubation with NaOCl. This removes the smear layer, giving NaOCl improved access to the dentinal tubules. However, in accordance with previous reports [15, 16], our results demonstrated that smear layer removal by this method does not increase the antimicrobial effect of NaOCl.

In recent years, several studies have focused on evaluating the effectiveness of BioPure MTAD as a root canal irrigant against *E. faecalis*. Newberry et al. [26] determined the antimicrobial effect of BioPure MTAD as a final irrigant on eight strains of *E. faecalis*. After irrigating with 1.3% NaOCl, the root canal and external surfaces were exposed to BioPure MTAD for 5 min. Roots or dentin shavings were cultured to determine the growth of *E. faecalis*, and results showed that this treatment regimen was completely effective at eliminating the growth of seven of the eight *E. faecalis* strains. Furthermore, Davis et al. [27] used in vitro experiments to show that 2% chlorhexidine and 5.25% NaOCl both exhibited less antimicrobial efficacy against *E. faecalis* than BioPure MTAD, demonstrating that BioPure MTAD is a viable medicament against *E. faecalis*. In another study, Mohammadi and Shahriari [28] compared the antimicrobial effect of Biopure MTAD, 2% chlorhexidine and 2.6% NaOCl on *E. faecalis* in human root dentin. Their findings showed the BioPure MTAD was more effective than the other solutions, and was retained in the root canal dentin for at least 28 days. These findings are consistent with our own results (Group C) and those of other researchers [15, 16, 29–32] who have reported the superior efficacy of BioPure MTAD against *E. faecalis*.

However, our results are contrary to some other reports on the antimicrobial effects of irrigants. A study by Kho and Baumgartner [20] compared the antimicrobial efficacy of irrigation with 1.3% NaOCl/Biopure MTAD with that of irrigation with 5.25% NaOCl/15% EDTA in the apical 5 mm of roots infected with *E. faecalis*, and found no difference between these treatments. In addition, Baumgartner et al. [21] found no growth of *E. faecalis* in root canals irrigated with 5.25% NaOCl/15% EDTA, while 50% of the canals irrigated with 1.3% NaOCl/Biopure MTAD demonstrated growth of *E. faecalis*, a difference that was statistically significant. Our results are also in disagreement with those of Dunavant et al. [19], Giardino et al. [33], Clegg et al. [34], Ruff et al. [35], and Krause et al. [36], each of whom showed that NaOCl was more effective than BioPure MTAD at eliminating *E. faecalis*. These differences ensure that the efficacy of BioPure MTAD remains somewhat controversial, although are probably in part explained by methodological differences such as alternative microbial sampling procedures or deviation from the manufacturer's usage recommendations when using BioPure MTAD.

## 5. Conclusions

Our findings suggest that BioPure MTAD possesses superior bactericidal activity compared with NaOCl and EDTA against *E. faecalis* in contaminated root canals. However, further clinical studies are required to confirm the in vivo antimicrobial effects of this and other endodontic medicaments.

## Figures and Tables

**Table 1:** Irrigation protocol.

Group	No. of teeth	Irrigation during cleaning and shaping	Final rinse	Immersion
Positive control	6	Distilled water	dH_2_O	dH_2_O
Negative control	6	Distilled water	dH_2_O and autoclave	dH_2_O
A	6	1.5% NaOCl, 1 mL after each file	1.5% NaOCl, 5 mL	1.5% NaOCl, 5 min
B	6	3% NaOCl, 1 mL after each file	3% NaOCl, 5 mL	3% NaOCl, 5 min
C	6	1.5% NaOCl, 1 mL after each file	MTAD, 5 mL	MTAD, 5 min
D	6	1.5% NaOCl, 1 mL after each file and EDTA 17%, 1 min	1.5% NaOCl, 5 mL	1.5% NaOCl, 5 min
E	6	3% NaOCl, 1 mL after each file and EDTA 17%, 1 min	3% NaOCl, 5 mL	3% NaOCl, 5 min

**Table 2:** Presence of *E. faecalis* in tooth samples treated with different endodontic irrigants.

Groups	No. of Samples	Growth		
		One week	Dentin shavings	Total
Positive	6	6	6	6
Negative	6	0	0	0
A	6	5	1	6
B	6	2	1	3
C	6	0	1	1
D	6	5	1	6
E	6	5	1	6
